# Venoarterial Extracorporeal Membrane Oxygenation as A Bridge to Surgery in Post-Myocardial Infarction Ventricular Septal Defect with Cardiogenic Shock: Case Report

**DOI:** 10.21470/1678-9741-2021-0151

**Published:** 2023

**Authors:** Santiago Besa, Jonathan Walbaum, Rodrigo González, Fernando Baraona, Luis Garrido-Olivares

**Affiliations:** 1 Cardiovascular Surgery, Division of Surgery, Pontificia Universidad Católica, Santiago, Chile.; 2 Medical Student, Pontificia Universidad Católica, Santiago, Chile.; 3 Division of Cardiovascular Disease, Pontificia Universidad Católica, Santiago, Chile.

**Keywords:** Extracorporeal Membrane Oxygenation, Ventricular Heart Septal Defects, Shock, Cardiogenic, Myocardial Infarctation, Hospital Mortality

## Abstract

We describe a 60-year-old woman with post-myocardial infarction (MI) ventricular septal defect (VSD) and cardiogenic shock who was successfully stabilized with veno-arterial extracorporeal membrane oxygenation (VA-ECMO) as a bridge therapy for the surgical closure of her VSD. This case highlights the role of VA-ECMO in the management of post-MI VSD to improve the results of surgical repair and patient survival.

## INTRODUCTION

Post-myocardial infarction (MI) ventricular septal defect (VSD) is a rare mechanical complication that occurs in <1% of MI cases with mortality >90% if treated medically and 15-60% with surgical intervention^[1]^. The most important risk factors include age, anterior wall MI and female sex, while current smoking and prior MI are protective factors^[2]^. It is a medical emergency associated with cardiogenic shock, multiple organ failure and death^[3]^. Immediate surgical closure is recommended as it drastically reduces mortality, but better surgical outcomes are obtained if delayed, as it allows hemodynamic stability, healing and organization of friable myocardial tissue^[3,4]^. A proposed technique to delay surgical intervention is the use of preoperative venoarterial extracorporeal membrane oxygenation (VA-ECMO), which, according to the Extracorporeal Life Support Organisation (ELSO), is indicated in acute heart failure potentially reversible and unresponsive to conventional management, among other situations^[5]^. In this context, acute heart failure due to post-MI VSD is an indication for ECMO support. We present a case in which VA-ECMO was successfully used as bridge therapy to surgery in a patient with cardiogenic shock associated with post-MI VSD.

### Case Report

A 60-year-old woman with a history of hypertension and type 2 diabetes with no treatment for the past year was admitted to a regional hospital after more than 12 hours of oppressive chest pain and dyspnea. Blood pressure was 100/58 mmHg, oxygen saturation 90% and heart rate 98 beats/min. Holosystolic murmur at the apex and bilateral crackles were present.

**Fig. 1:**
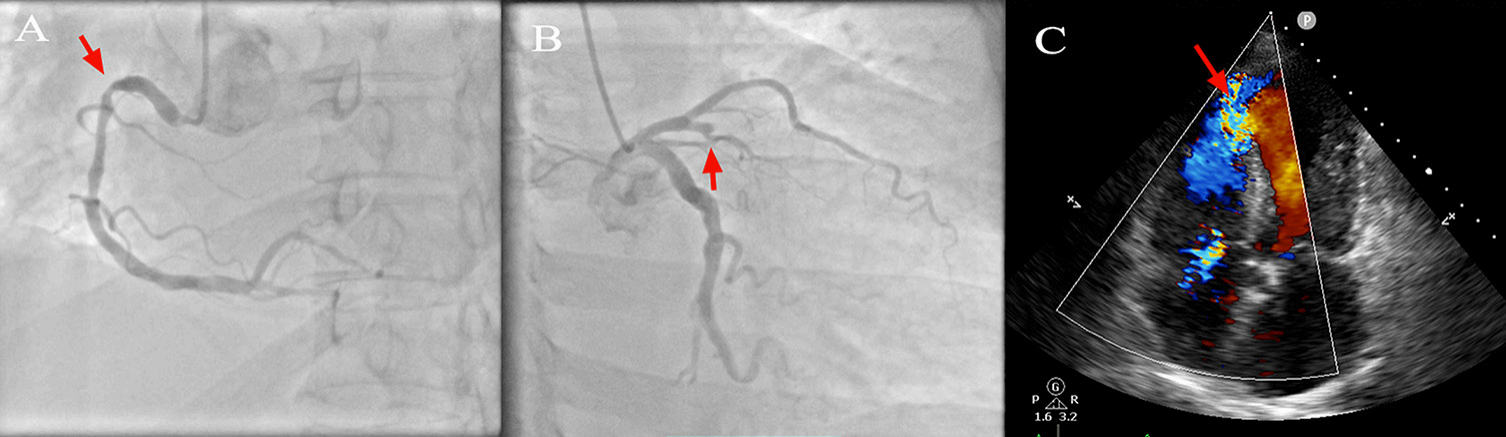
Diagnostic imaging. (A) Coronary angiography showing 80% obstruction of RCA (arrow) and (B) complete obstruction of the ADA (arrow). (C) 2D color echocardiographic image of apical VSD (arrow).

The electrocardiogram (ECG) showed an extensive anterior wall ST segment elevation compatible with MI. After failed thrombolysis with tenecteplase (no changes in ECG), the patient was transferred to another institution for a more complex care. Twenty-four hours after emergency room (ER) consultation, coronary angiography showed severe two-vessel disease with complete obstruction of proximal anterior descending artery (ADA) and 80% obstruction of proximal right coronary artery (RCA). Percutaneous angioplasty of ADA was unsuccessful. An apical VSD was discovered during the procedure. Echocardiography confirmed the VSD, with left ventricular ejection fraction (LVEF) of 40% (Figure 1, Videos 1 and 2). Therefore, the patient was transferred to our center for coronary artery bypass grafting surgery and VSD repair.

**Video 1:**
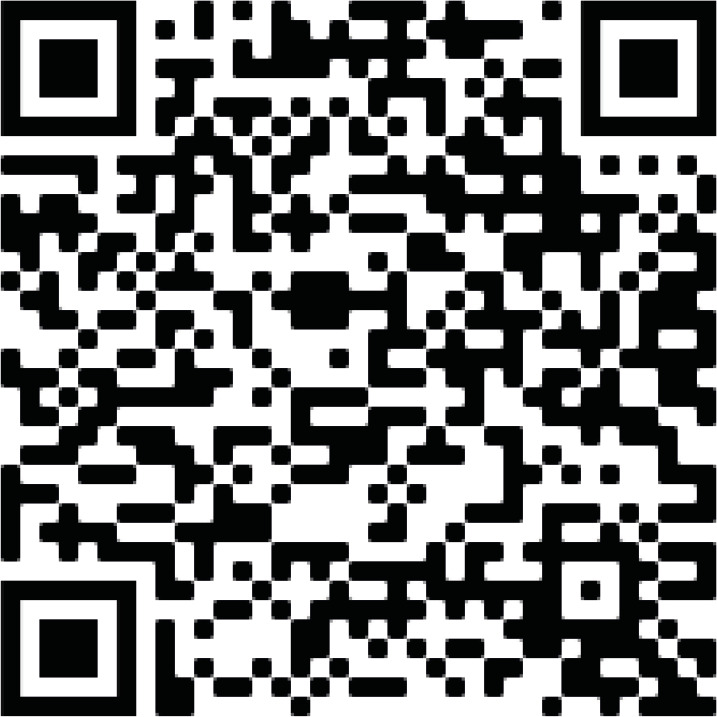
LV ventriculogram. After contrast injection, a septal-apical VSD is revealed. Estimated LVEF of 40%.

**Video 2:**
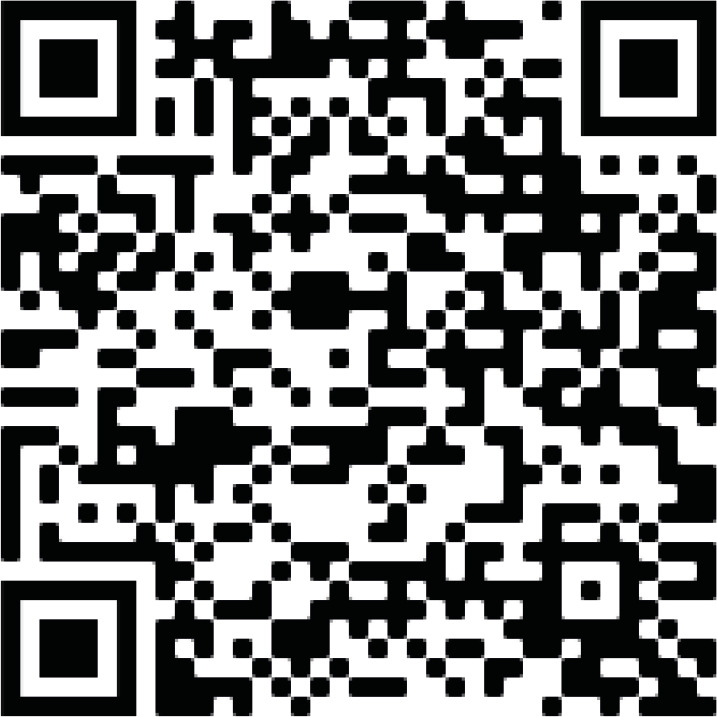
2D color echocardiogram. The apical 4-chamber view shows a non-restrictive VSD with left-to-right flow.

**Fig. 2:**
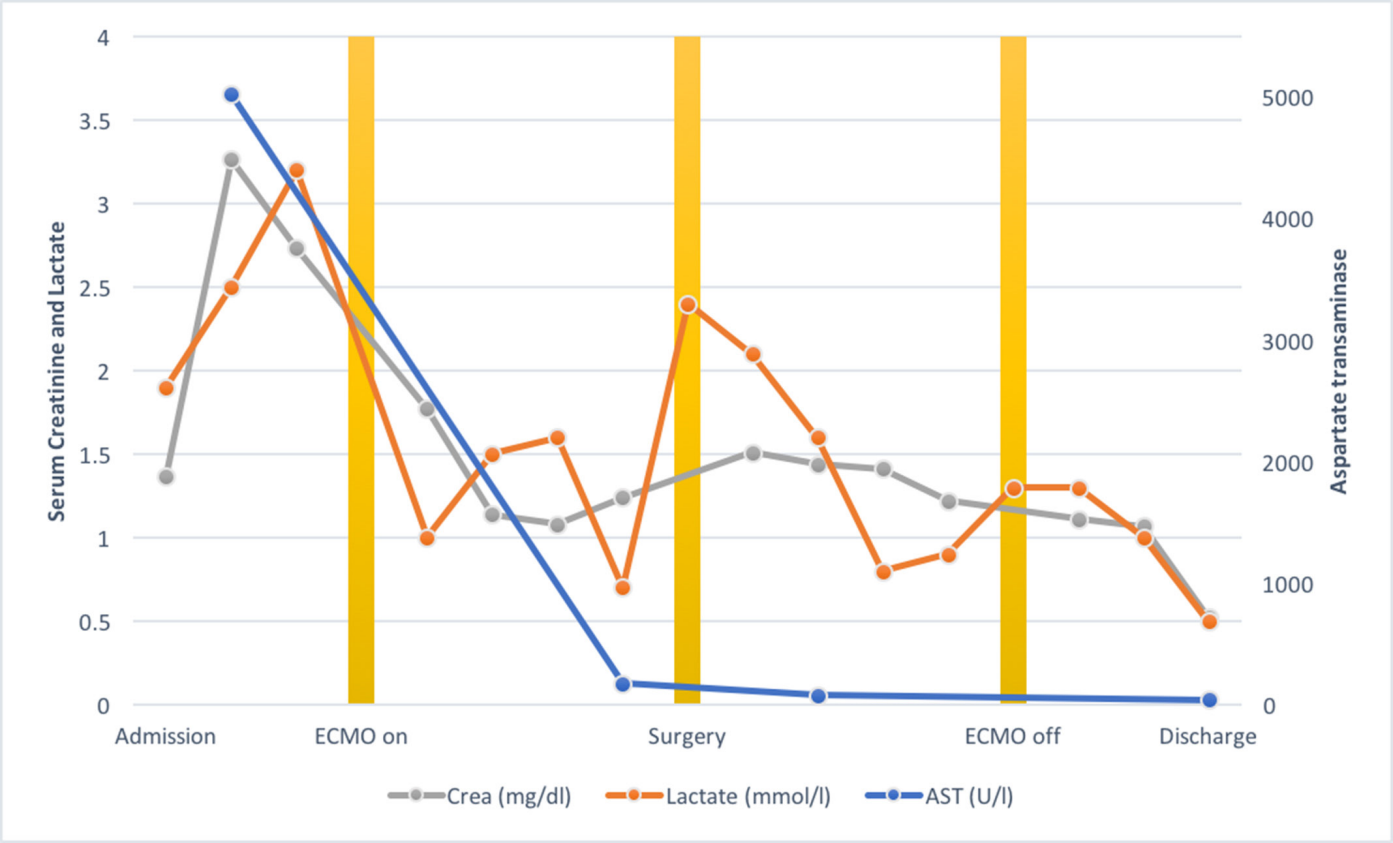
Laboratory parameters during hospitalization. Evolution of serum creatinine, lactate and aspartate transaminase levels is shown before, during, and after ECMO support. ECMO connection led to an improvement in all parameters.

The patient was admitted to our service 36 hours after consultation in cardiogenic shock (cardiac index = 1.2 l/min/m^2^) with evidence of renal and hepatic dysfunction and high-dose vasoactive support. The VA-ECMO was established through femoral access. Hemodynamic stability was achieved with suspension of vasoactive drugs within 48 hours and normalization of both renal and hepatic function within 4 days (Figure 2). Echocardiography during ECMO did not show left ventricle (LV) or right ventricle (RV) dilatation and chest radiography did not show pulmonary edema, thus no additional LV unloading device or venous drainage cannula were necessary. On day 5 of VA-ECMO, VSD was repaired by infarct exclusion using the David technique^[1]^, along with a saphenous vein bypass graft to the RCA without incident. Both femoral arterial and venous ECMO cannulas were used for cardiopulmonary bypass (CPB) by adding only a superior vena cava cannula. ECMO circuit was kept circulating through a shunt during CPB and was reused after weaning from CPB. The patient was kept on VA-ECMO and gradually weaned, until successful disconnection on day 9. Weaning from invasive mechanical ventilation was achieved 48 hours after VA-ECMO disconnection.

On day 15, echocardiography showed a closed VSD and akinesia of the apex, mid-anteroseptal segment and all apical segments of the LV and LVEF of 40%. The patient was successfully discharged home 28 days after surgery.

**Fig. 3:**
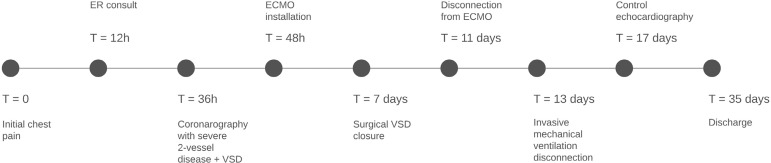
Summary timeline from the onset of patient’s chest pain until discharge. The time of main events and interventions is shown.

A timeline of patient evolution is detailed (Figure 3).

## DISCUSSION

Post-MI VSD is a rare complication of MI (<1% of cases) with mortality >90% if treated medically and 15-60% with surgical intervention^[1]^. Incidence has decreased over the years as stated in the GUSTO-I trial, which could be attributed to more accessible early reperfusion therapy. Important risk factors for post-MI VSD include advanced age, anterior location of the infarction and female sex. Patients who develop post-MI VSD are more likely to have complete occlusion of the affected artery, which suggests that the pathophysiology of acute VSD involves sudden, severe ischemia and subsequent extensive necrosis, with increased risk in patients in whom early reperfusion is not successful^[2]^. It is a medical emergency associated with cardiogenic shock, multiple organ failure and death^[3]^. Immediate surgical closure is recommended as it significantly reduces mortality, but better surgical outcomes are obtained if it is delayed, as this allows for hemodynamic stability, healing and organization of friable myocardial tissue^[3,4,5]^. A technique proposed to delay surgical intervention is the use of preoperative VA-ECMO, which, according to ELSO, is indicated in acute heart failure, potentially reversible and unresponsive to conventional management, among other situations^[6]^. We believe that ECMO support is the best tool available to rescue a patient from cardiogenic shock, allowing for stabilization and improvement of all organ functions and for non-emergent, but planned, surgical repair.

Mortality associated with surgical repair of post-MI VSD varies depending on the time of surgery, . A retrospective review using data from The Society of Thoracic Surgeons reported a mortality rate of 54.1% with early intervention (<7 days) and 18.4% with late intervention (>7 days)^[1]^. Omar et al.^[3]^ describe that surgical repair and transcatheter closure (TCC) have a significantly lower mortality than medical management alone, 92% *versus* 61% respectively, and there is no difference in mortality between early TCC and early surgical repair. Shock at time of operation and time from infarction to surgery were the only significant independent predictors of mortality in a study conducted by Deja et al.^[4]^.

With respect to the surgical technique, infarct exclusion as described by David et al.^[7]^ is associated with lower mortality and a non-significant difference in mortality between anterior and posterior VSD^[4]^. Surgical treatment with infarct exclusion and patch repair has been described as the treatment of choice in these patients^[6]^.

Guidelines published in 2013 stated that emergency surgical repair is recommended in all VSD patients, regardless of their preoperative hemodynamic status, but an update of these guidelines in 2017 recommended that delayed surgery could be considered in patients who respond well to aggressive heart failure therapy^[5]^.

Ventricular assist devices (VADs) have been used to decrease preload and afterload and allow myocardial rest and healing. This can be used both pre- and postoperatively, ensuring organ perfusion in the presence of a damaged heart, allowing relative recovery of organ function and clinical stability^[8]^. It has the potential to allow patients in refractory cardiogenic shock to stabilize and undergo surgical VSD repair in more favorable conditions, with better surgical outcomes when surgery is delayed and decreasing the rate of recurrence and dehiscence^[3,4,5,8,9]^.

ECMO has many advantages over other VADs, such as its relatively easy implantation, complete cardiopulmonary support and relatively low anticoagulation requirements^[8,10]^. On the other hand, despite providing RV unloading, it produces an increase in afterload that can impair LV unload for rest. LV distension is somewhat attenuated by the same VSD that works as a natural LV unloading port, but at the expense of increased left-to-right shunt and RV overload with subsequent worsening of pulmonary edema^[11]^. Nevertheless, there are strategies to improve the hemodynamic effect of ECMO support and address the undesired effects. Adding an unloading device such as an intra-aortic balloon pump or Impella™ would reduce LV afterload and decrease shunt. Adding a venous drainage cannula would resolve the RV volume overload. A pulmonary artery cannula would achieve both mechanisms at the same time, indirectly venting the LV and managing the RV overload^[12]^. None of these strategies were necessary for this patient, possibly because the systemic venous drainage was enough to manage the right overload, as would be expected in many of these patients. Furthermore, ECMO has been shown to be effective in maintaining optimal end-organ perfusion and gas exchange, thus facing the effects of severe pulmonary congestion secondary to shunt^[13]^.

As stated before, timing of definitive treatment must be carefully decided case-by-case, considering risk against benefit, as prolonged ECMO increases the risk of complications including coagulopathy, bleeding and infections, possibly compromising the benefit of delaying surgery^[9]^. In a recent series of 8 patient cases, patients connected to ECMO before surgical intervention significantly improved end-organ perfusion biomarkers, demonstrating better organ function at the time of surgery. This may have contributed to better short-term surgical outcomes^[5]^. In this study, surgical intervention was performed as soon as hemodynamic and organ function stabilized to prevent complications associated with ECMO described above, but it was concluded that there is no consensus regarding the time of intervention and further studies should be carried out^[5]^.

## CONCLUSION

Post-MI VSD is a life-threatening complication of myocardial infarction and requires surgical or percutaneous repair. Surgical results and survival can be drastically improved by previous stabilization of the patient with the use of VA-ECMO.
